# Factors Affecting Consumers’ Online Choice Intention: A Study Based on Bayesian Network

**DOI:** 10.3389/fpsyg.2021.731850

**Published:** 2021-10-20

**Authors:** Weibin Deng, Ting Su, Yiming Zhang, Chunli Tan

**Affiliations:** ^1^Key Laboratory of Electronic Commerce and Modern Logistics, Chongqing University of Posts and Telecommunications, Chongqing, China; ^2^School of Economics and Management, Chongqing University of Posts and Telecommunications, Chongqing, China; ^3^78111 Troops, People’s Liberation Army of China, Chengdu, China

**Keywords:** two-stage decision-making theory, search intention, purchase intention, structural equation model, Bayesian network

## Abstract

In China, the mature development of online retail channels provides consumers with multiple consumption choices, and the factors that affect whether consumers choose to search or purchase online are numerous and complex. In this context, this paper reports on experimental research regarding consumers’ willingness to choose channels based on the two-stage decision-making theory. Using structural equation modeling, the factors influencing consumers’ online search intention and purchase willingness and the relationship between them are studied. In particular, the perceived benefits, channel trust, and channel transfer costs are explored. Furthermore, a Bayesian network is used in order to analyze the degree of influence of each factor quantitatively. It is found that online trust is an important factor affecting consumers’ online search intention, and the most important factor for consumers’ online purchase intention is their perceived benefits of online shopping. At the same time, there is a positive relationship between online search intention and purchase intention. This study can provide management decision support for online retail enterprises and help to promote the healthy development of online shopping.

## Introduction

The recent emergence of multiple retail channels has made consumers’ choice of shopping channels more complex, causing consumers to rethink their choice of shopping channels. This phenomenon has attracted the attention of scholars, who have examined product pricing and channel choice willingness. Many scholars have analyzed channel choice willingness from the theoretical perspective of consumer perception, as consumer behavior is motivated by consumers’ psychological assessment of the results that will be achieved by the specific attributes of products or services, such as perceived benefit (Khan et al., 2015), perceived value (Zhao and Chen, 2021), and perceived usefulness (Wang et al., 2021). However, the above-mentioned researches are one-sided, because they only analyze the choice of shopping channels from the perspective of consumer perception. Trust is the attitude and cognition of consumer toward shopping channels. It is believed by some scholars that perceived benefits are based on trust toward the shopping channels (Costa e Silva et al., 2020). Both online and offline channels boast their own advantages, and consumers can choose different channels at different purchasing stages. But current researches can hardly clarify the complexity on studying consumer channel choice in a theoretical way.

Consumer channel choice is the study of consumer behavior with unique features, because it is based on analyzing real problems and giving choices. The early researches are dominated by theoretical analysis. For example, Huang et al. (2016) analyzed the impact exerted by the emergence of mobile retail channels on online consumption behavior. Based on qualitative research, some scholars try to adopt statistical methods and models to study consumer shopping channels, such as using correlation analysis to analyze factors influencing consumers in online shopping decision-making (Elida et al., 2019). However, the relationship between variables cannot be well explained and the latent variable measurement error remains unresolved by the mentioned methods. With the development of statistical theory, some more rigorous and sound statistical techniques and model analysis methods have been introduced into the research on factors affecting online shopping, such as research on impulsive consumption in online retail (Gupta and Shukla, 2019) and the influence of brand experience on consumer behavior (Chen-ran, 2020). Most of the above researches are carried out around the structural equation model (simply called SEM). SEM is a multivariable statistical analysis method for testing the hypothetical relationships between observed variables and latent variables and among latent variables. It has good processing ability in proving the authenticity of hypotheses (Akbarzadeh et al., 2019). In the behavior research of online consumer, latent variables, such as cognition, attitude, behavior, and willingness, are often unmeasurable, which need to be represented by observed variables. By combining the characteristics of the online consumer behavior, the SEM pre-selects several factors that affect the consumption behavior, sets up the relevant observation and latent variables, and builds the path analysis model. In the research, it is positive to observe the multiple relationships between different variables by considering the significance, coefficient, and mediation or moderating effects to determine the variables correlation. However, building the entire path analysis framework relies on subjective assumptions and judgments. Setting different paths will correspondingly produce different results, therefore, and it is difficult to ensure its stability. Such research is the confirmatory research and highly related to research hypotheses, which greatly limits how this method is applied in investigating online consumer behavior. At the same time, the SEM lacks the ability to predict and diagnose the relationship between variables (Song and Lee, 2008). A Bayesian network is a statistical method for expressing the causality between variables and the relationship between prediction and diagnosis variables (Chickering, 2002). Due to its good prediction and diagnosis ability, it can be used to accurately analyze consumers’ purchasing behavior (Song et al., 2013). However, it lacks the empirical ability of examining variable relationships (Song et al., 2011). Therefore, this paper proposes to combine SEN and Bayesian network, which not only adopts SEM in the empirical research to fit non-standard models, but also uses Bayesian network to make diagnosis and prediction. Based on two-stage decision-making theory, we take into account the online channel searching and purchasing intention of consumers in this paper and accurately analyze the factors that affect how consumers make choices online and their complex relationships. It can provide references for online retail companies to formulate reasonable marketing strategies.

## Theory and Hypotheses

### Two-Stage Decision-Making Theory

Once consumers generate a shopping desire, searching for information and buying products are the most two important stages of their shopping decision-making process. Haubl and Trifts (2000) presented a two-stage decision-making theory based on the study of consumer shopping behavior. In the search stage, consumers search for a large amount of relevant information about the product. In the purchase stage, they make an in-depth comparison and evaluation of the options, and then, they make the final purchase decision. Two-stage decision-making theory has been applied by many scholars in the choice of consumption channels. Schneider and Zielke (2020) used two-stage decision-making theory to study the consumer Showrooming behavior. Meanwhile, Balladares et al. (2016) studied the factors that affect consumers in the information search stage based on two-stage decision-making theory. Singh and Jang (2020) studied the impact of consumer’s perception on choosing searching and purchasing channels and the satisfaction.

Due to the coexistence of online and offline retail channels, consumers have more choices in purchasing channels, and channel choice willingness is the main factor for measuring consumers’ channel choice behavior, because consumers have different channel selection behaviors when they are in different purchase decision-making stages. Hence, we can get four consumers’ channel choice models: search online–purchase online, search online–purchase offline, search offline–purchase online, and search offline-purchase offline. Based on this, this paper discusses the factors that affect consumers’ online search and purchase intention and the complex relationship between these intentions based on two-stage decision-making theory.

### Bayesian Network

**Figure 1:**
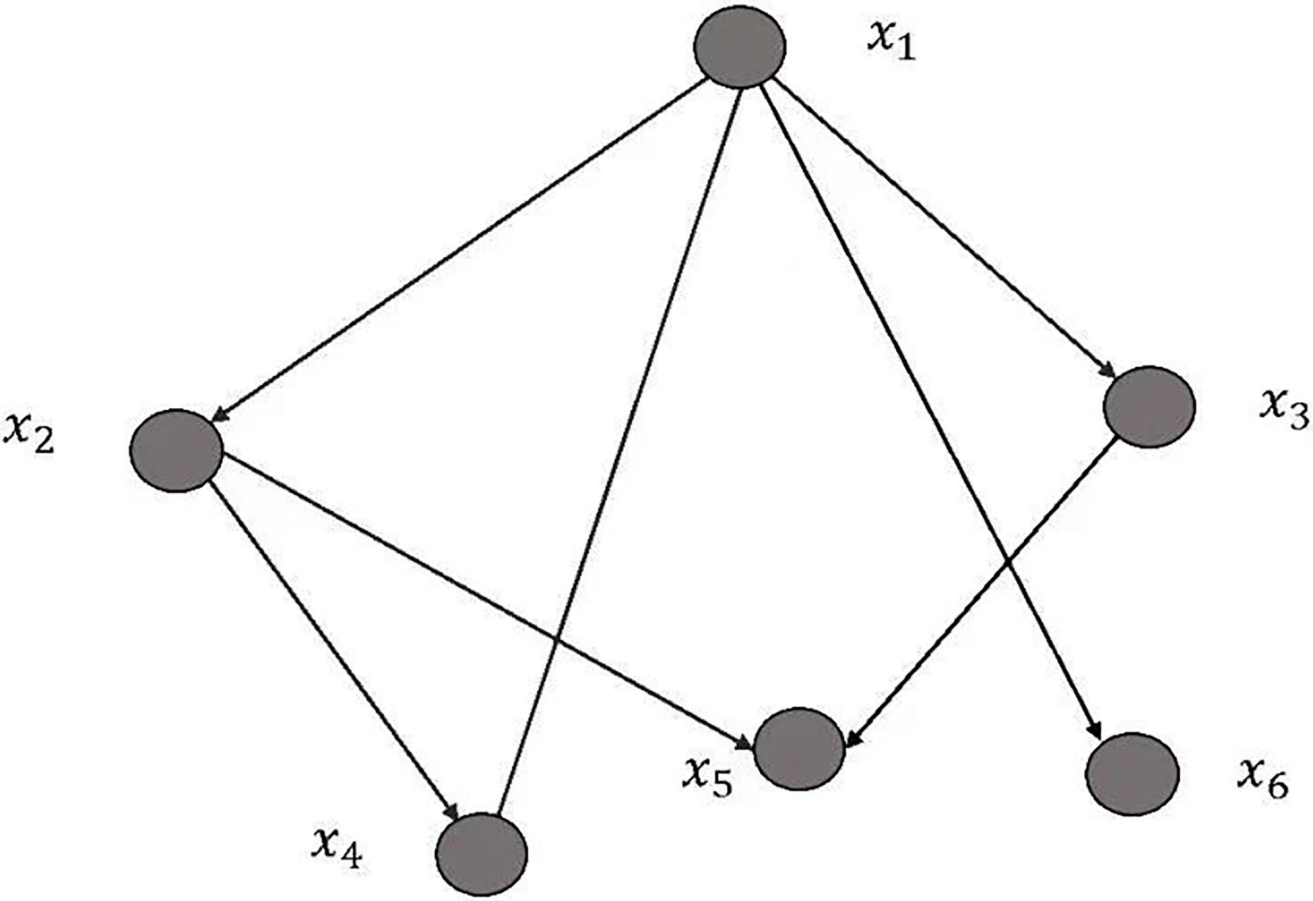
Bayesian network structure.

A Bayesian network shows the relationship between latent variables in the form of a causality graph, which is composed of a network structure *S* and parameter set *θ*. The network structure *S* is used to represent the independent and conditional independent relationship between the sets of classified random variable *x*={*x*_1_, *x*_2_,…, *x_n_*}, and the network structure *S* is composed of nodes and directed arcs, which is a directed acyclic graph. The parent node of the node *x_i_* is represented by *pa_i_*, and the value set of the parent node is represented by the value set of the parent node: $pa_{i}=\left\{pa_{i}^{1},pa_{i}^{2},\ldots ,pa_{i}^{r_{pa_{i}}}\right\}.$ The parameter set *θ* is the local probability corresponding to each variable, and it is the conditional probability set under a given parent node. The parameter set of the variable *X_i_* is as follows: $\theta_{x_{i}}=\left\{P\left(x_{i}^{1}|pa_{i}^{j}\right),P\left(x_{i}^{2}|pa_{i}^{j}\right),\ldots ,P\left(x_{i}^{r_{i}}|pa_{i}^{j}\right)\right\}.$ $j=1,2,\ldots ,r_{pa_{i}}.$ Figure 1 shows the Bayesian network structure.

There are many Bayesian network algorithms. The TAN (Tree-Augmented-Naive) Bayesian network proposed by Friedman et al. relaxes the application conditions of the classic Bayesian network and allows complex correlations between variables. TAN Bayesian networks are trained by constantly training the sample sets to find the best parameters *S*, *θ*, which is also the analysis method used in this article. TAN Bayes is an extension of the classic Bayesian network model. It can handle variables that have correlations and have good predictive power for high-dimensional data. The basic idea of the TAN Bayesian network is to use the Bayesian network to express the dependency relationship and to connect the relationship between attribute variables with a directed arc from the parent node to the child node. TAN Bayesian networks are widely used in data mining in the fields of computer, business and communication.

The Bayesian model involves the causal prediction and inference of the observed variables, while the SEM involves empirical analysis of the path relationship of the latent variables. Therefore, the key to combining the SEM and Bayesian network is to obtain the sample data of each node of the Bayesian network through the observation variables to make predictions and diagnostic analysis. The main design ideas of this paper are as follows:

First, we identify the factors influencing consumers’ online choice, as shown in Table 1, collect data through a questionnaire survey, and then construct the relationship between the observed variables of the SEM.

**Table 1:** List of variables, measure items, and literature sources.

Variable	Items	Sources
Perceived benefit (PB)	Online channels can provide detailed product information.	Verhoef et al., 2007
	Online channels can provide a wide range of products to meet my diverse needs.	
	Online channel can easily compare the same type of products.	
	I can get product information quickly through online channels.	
	The online channel purchase process is easy to operate and easy to purchase.	
	There are many discounts in online channels.	
Channel trust (CT)	Other consumers’ evaluations of the product are reliable.	Kuan and Bock, 2007
	The seller’s reputation is trustworthy.	
	The quality of the product is reliable.	
	Merchant service is trustworthy.	
Switching cost (SC)	I need to spend extra money.	Burnham et al., 2003
	I need to spend extra time.	
	I need to spend extra energy.	
	It will bring me inconvenience.	
Online search intention (OSI)	I will recommend to the people around me to search for products through online channels.	Holloway et al., 2005
	I browse the shopping website every day.	
	I rely on shopping websites to search for products.	
	It is attractive for me to search for products through online channels.	
Online purchase intention (OPI)	I will shop online in the future.	Holloway et al., 2005
	Online shopping is my first way of shopping.	
	I am willing to buy more products through the shopping website.	
	I would like to recommend to the people around me to use the shopping website to buy products.	

Second, based on the SEM, we lay the foundation for the construction of the Bayesian network by calculating the score of each latent variable in the SEM.

Third, based on the relationship between latent variables in the SEM and the score of latent variables, in order to draw better research conclusions, the Bayesian network is used to further predict and diagnose the relationship between variables.

### Hypotheses

#### Search Intention

Searching is an important part of consumers’ purchase decision-making stage; the more abundant product information consumers have, the more likely they are to make satisfactory purchase decisions, but their willingness to engage in the information search is limited by the cost of the channel search (Tien and Kiureghian, 2016). From the perspective of utility maximization, consumers will choose the lowest-cost way to search for product information. Compared with completing purchases through multiple channels, consumers will spend less in a channel to search for information and buy the products. It is indicated by Singh and Swait (2017) that online channels provide greater searching or purchasing benefits. Ngwe et al. (2019) found that guiding consumers to search for products will increase the overall expected purchasing probability of sold products. Zhai et al. (2019) showed that the channel searching and purchasing behavior of consumers can influence each other.

Trust transfer theory is widely used in the study of consumer behavior when multiple retail channels coexist. Scholars divide trust transfer into intra-channel and inter-channel trust transfer (Stewart and Qin, 2013). Intra-channel trust transfer refers to consumers’ trust transfer between different shopping stages in the same channel (online or offline; Lee et al., 2011). Path dependence theory points out that once economic, social, or technological systems enter a certain path, for better or worse, they will constantly strengthen themselves under the action of inertia (Thietart, 2015). In other words, people’s past choices determine their possible choices now. When consumers enter the online retail environment, the impact of search willingness on purchase intention is also a manifestation of path dependence. Based on the theories of trust transfer and path dependence within the channel, consumers’ willingness to search in one channel affects their willingness to buy in the same channel. Therefore, the first hypothesis is proposed:

*H1*: There is a positive relationship between online search intention and purchase intention, and purchase intention can have a reverse impact on search intention.

#### Perceived Benefit

Both online and offline channels have the functions of information search and product sales (Balasubramanian et al., 2010). However, because different channels have different characteristics, consumers have different perceived benefits of product selection, product quality, service quality, and so on, which will affect their choice of channels. According to Lee et al. (2018), perceived benefit is the customers’ evaluation of the overall utility of using a certain channel based on their own needs, which has a direct impact on their purchase decisions. Due to the particularity of online channels, consumers cannot personally experience the utility of products when shopping through such channels. When consumers make an evaluation, one of the most direct factors to consider is the benefits that the channels can bring; the greater the perceived benefits, the stronger the consumers’ willingness to buy the products (Martin et al., 2015). van der Lans et al. (2016) pointed out that perceived benefits are most important in determining purchase intentions. The perceived benefits of channels not only affect consumers’ willingness to purchase but also attract consumers’ willingness to search. Based on the above analyses, the degree of consumers’ perceived benefits of retail channels reflect their willingness to choose search information or purchase products. Building on this discussion, the study suggests the following hypotheses:

*H2*: There is a positive relationship between perceived benefits and online search intention, and search willingness can have a reverse impact on perceived interests.

*H3*: There is a positive relationship between perceived benefits and online purchase intention, and purchase intention can have a reverse impact on channel trust.

#### Channel Trust

Many scholars have proved that trust is one of the main factors affecting consumers’ intention to purchase, especially when they cannot touch the transaction object as in the online retail environment, consumers will rely on trust to reduce the uncertainty of their purchase decisions, hence increasing the probability of interaction between consumers and retail channels. Channel trust is a reliable way for consumers to search for information. Consumers will trust the channel more because of its reliability and the high-quality information it provides. Trust will increase consumers’ goodwill toward businesses and reduce their perceived risks (Zhao et al., 2017). In a study of consumer behavior, Martin et al. (2015) found that consumer trust has a positive impact on channel choice intention. Reimer and Benkenstein (2016) studied the impact of the credibility of other online consumers’ comments on consumers’ channel choices. They found that the higher the consumers’ trust in the channel, the more likely they are to think that online reviews are more credible. Hajli (2015) argued that consumers’ trust and purchase willingness are affected significantly by online retailers’ ratings and comments, recommendations and introductions, and forums and communities. King et al. (2014) showed that consumers’ trust for a certain brand or product significantly affects their purchase willingness. Hence, the following hypotheses are presented:

*H4*: There is a positive relationship between channel trust and online search intention, and search intention can have a reverse impact on channel trust.

*H5*: There is a positive relationship between channel trust and online purchase intention, and purchase intention can have a reverse impact on channel trust.

#### Switching Cost

In the multi-channel retail environment, consumers’ consumption behaviors are different online and offline, and the switching cost is the additional cost that consumers must pay for switching services. It includes the economic, psychological, and even emotional cognitive costs of stopping the use of current services and changing to new ones. The switching cost is the multi-channel consumers’ perception of the time and energy spent on the conversion between offline and online channels, and it is a part of their assessment of the total shopping cost. Some scholars (Anderson and Simester, 2013; Stan et al., 2013) have shown that the switching cost has a significant impact on the choice of consumer information search channel and purchase channel in the multi-retail channel environment. Specifically, the higher the switching cost, the less easy it is for consumers to make cross-channel purchases. It is discovered by the research of Chang et al. (2017) that the switching cost prevents free-riding behavior. In this paper, the switching cost is set as the perceived cost caused by consumers transferring from the online channel to the offline channel. Building on this discussion, the next set hypotheses are stated as follows:

*H6*: There is a positive relationship between switching cost and online search intention, and search intention can have a reverse impact on switching cost.

*H7*: There is a positive relationship between switching cost and online purchase intention, and purchase intention can have a reverse impact on switching cost.

**Figure 2:**
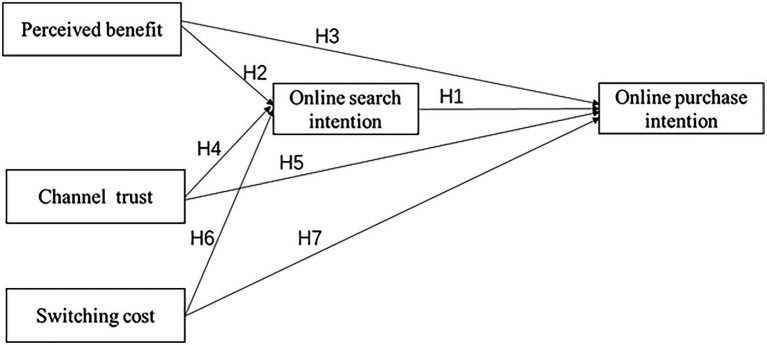
Conceptual model of consumers’ intention to choose channels.

Based on the above assumptions, the conceptual model is shown in Figure 2.

## Methods and Results

### Data Collection and Sample

A five-level Likert scale is used, with options of “very much agree,” “agree,” “generally agree,” “disagree,” and “very much disagree,” corresponding to values of 5, 4, 3, 2, and 1. The higher the degree of identity, the higher the score.

Before the formal survey, we conducted a pre-survey on the questionnaire with college students who have online shopping experience (100 students in total) and revised the questionnaire based on the suggestions made by them and experts. In order to ensure the randomness of the collected data, questionnaires are distributed through Internet after revised, targeting at consumers in China who have both online and offline shopping experience.

It takes a week to collect questionnaires. A total of 591 questionnaires are collected in total, 30 of which are invalid and thus excluded, reasons for invalidity included as: (1) answer time is not normal (e.g., answer time less than 30s), (2) have missing data on their questionnaires (e.g., the question “compared with offline channels, other consumers’ evaluation of the product is trustworthy” is not answered), and (3) have no obvious regular answers (e.g., choosing the same option for 10 or more successive questions). Finally, 561 questionnaires were actually processed, and the validity rate was 94.92%. There were more female participants (60.1%) than male participants (39.9%), including students (23.5%), office workers (7.0%), clerks (56.1%), and others (13.4%), and the possible explanation for imbalanced sex ratio is that women are more interested and enthusiastic in online shopping. Overall, 89.1% of the respondents were aged between 20 and 39years, and most were highly educated, including graduate (20.1%), undergraduate (65.2%), college degree (10.2%), and high school (4.5%). More detailed characteristics of the sample are shown in Table 2.

**Table 2:** Sample characteristics (*n*=561).

Item	Category	Percentage
Gender	Male	39.9
	Female	60.1
Age	<19years	3.0
	20–29years	59.7
	30–39years	29.4
	>40years	7.8
Education	High school	4.5
	College degree	10.2
	Undergraduate	65.2
	Graduate	20.1
Job	Student	23.5
	Office worker	7.0
	Clerk	56.1
	Others	13.4

### Reliability and Validity Test

We evaluated the reliability and internal consistency of the measure with SPSS 23.0. Cronbach’s alpha was calculated for the construct and ranged from 0.654 and 0.789, indicating that the reliability of each variable of the scale is acceptable and can be analyzed later. The reliability analysis results are shown as Table 3. In order to test the validity of the measured data, SPSS 23.0 was used to conduct an exploratory factor analysis. Principal component factor analysis of the data was carried out using the maximum variance method, and the results showed that the overall KMO (Kaiser-Meyer-Olkin) value of the factor analysis was 0.827 and the significance was 0.000, indicating that the data were suitable for factor analysis. According to the principle that the eigenvalue was greater than 1, five principal components were extracted, and the factor load of each measurement item was greater than 0.5, indicating that the measurement items of the unified construction variables were loaded on the same factor, and the scale had good convergence validity.

**Table 3:** Reliability analysis.

Variables	Items	Corrected item-total correlation	*α* if item deleted	Cronbach’s *α*
PI	PB1	0.522	0.775	0.798
	PB2	0.634	0.749	
	PB3	0.573	0.763	
	PB4	0.537	0.770	
	PB5	0.537	0.771	
	PB6	0.538	0.774	
CT	CT1	0.574	0.741	0.785
	CT2	0.609	0.723	
	CT3	0.643	0.705	
	CT4	0.542	0.757	
SC	CTC1	0.472	0.687	0.723
	CTC2	0.587	0.619	
	CTC3	0.577	0.630	
	CTC4	0.437	0.712	
OSI	OSI1	0.400	0.681	0.698
	OSI2	0.551	0.588	
	OSI3	0.566	0.577	
	OSI4	0.425	0.668	
OPI	OPI1	0.439	0.585	0.654
	OPI2	0.489	0.547	
	OPI3	0.458	0.572	
	OPI4	0.363	0.641	

**Figure 3:**
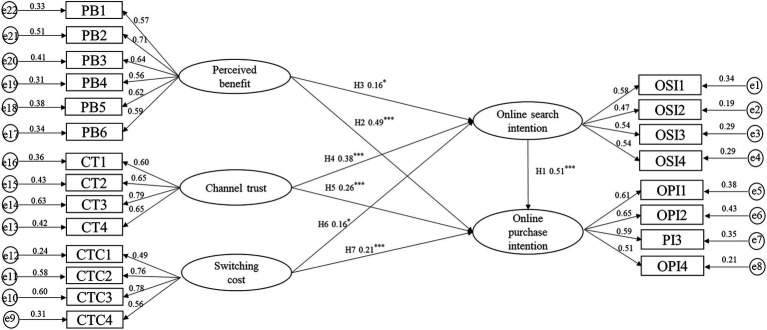
Path coefficient diagram of structural equation model.

In order to verify the scientific rationality of the model, it is necessary to test whether each fitting index meets the fitting standard. Take PB, OT, SC, SI, and PI as endogenous variables, and use Amos 23.0 to build the SEM shown as Figure 3. As shown in Table 4, the results of the fitting indexes in this study show a GFI (goodness of fit index) of model-fit of 0.969, CFI (comparative fit index) of 0.977, RMR (root mean square residual) of 0.029, X2/DF of 1.103, AGFI (adjust goodness of fit index) of 0.955, and RESEA (root mean square error of approximation) of 0.014. The fitting indexes meet the acceptance standard level.

**Table 4:** Index table of main fitting effects of SEM.

Fit Index	Statistics results	Standard line	Evaluation
RMR	0.029	<0.05	Meet the standard
X^2^/DF	1.103	<2.00	Meet the standard
GFI	0.969	>0.9	Meet the standard
AGFI	0.955	>0.9	Meet the standard
CFI	0.977	>0.9	Meet the standard
RMSEA	0.014	<0.05	Meet the standard

### Data Analysis With SEM

With the help of Amos 23.0, we used the maximum likelihood estimation method to verify the hypotheses proposed in this paper. It can be seen from the SEM that the influence of the variables is obvious. We can see that: (1) The path coefficients of the perceived benefit on online search intention and online purchase intention are 0.16 and 0.49, respectively, indicating that perceived benefit has a positive impact on search intention and purchase intention, and from the path coefficient, we can see that the perceived benefit has a greater influence on the purchase intention. (2) The path coefficients of channel trust on online search intention and purchase intention are 0.38 and 0.26, respectively, indicating that channel trust has a positive impact on search intention and purchase intention. (3) The path coefficients of switching cost on online search intention and purchase intention are 0.16 and 0.21, respectively, indicating that switching cost has a positive effect on search intention and purchase intention, and the influence on purchase intention is slightly greater than that on search intention. (4) The path coefficient of search intention on purchase intention is 0.26, indicating that search intention will also have a positive impact on purchase intention. Therefore, H1, H2, H3, H4, H5, H6, and H7 are supported.

### Data Analysis With Bayesian Network

The average score of each latent variable was analyzed by K-means cluster analysis with SPSS 23.0. In order to reduce the complexity of the operation and increase the identifiability of the judgment results, each latent variable was clustered into three states: high, medium, and low. Before clustering, in order to ensure the quality of the data, the box diagram of the sample data was drawn to deal with abnormal values, and the “minimum and maximum” abnormal data far away from the whole were eliminated; hence, a total of 42 outliers were removed, and 519 valid data were analyzed. In this study, an SPSS analysis of variance (ANOVA) was used to verify the differences in the latent variables in each dimension and to verify the significance of the classification to the dimension scores. The specific results are shown in Table 5. The ANOVA results show that it is reasonable to cluster sample data into high, medium, and low dimensions.

**Table 5:** Analysis of variance.

	Clustering		Error		*F*	Sig.
	Mean square	Df.	Mean square	Df.		
PB	4.789	2	0.146	516	32.696	0.000
CT	27.853	2	0.237	516	117.385	0.000
OSI	43.844	2	0.246	516	178.546	0.000
SC	19.638	2	0.409	516	48.019	0.000
OPI	9.428	2	0.140	516	67.262	0.000

**Figure 4:**
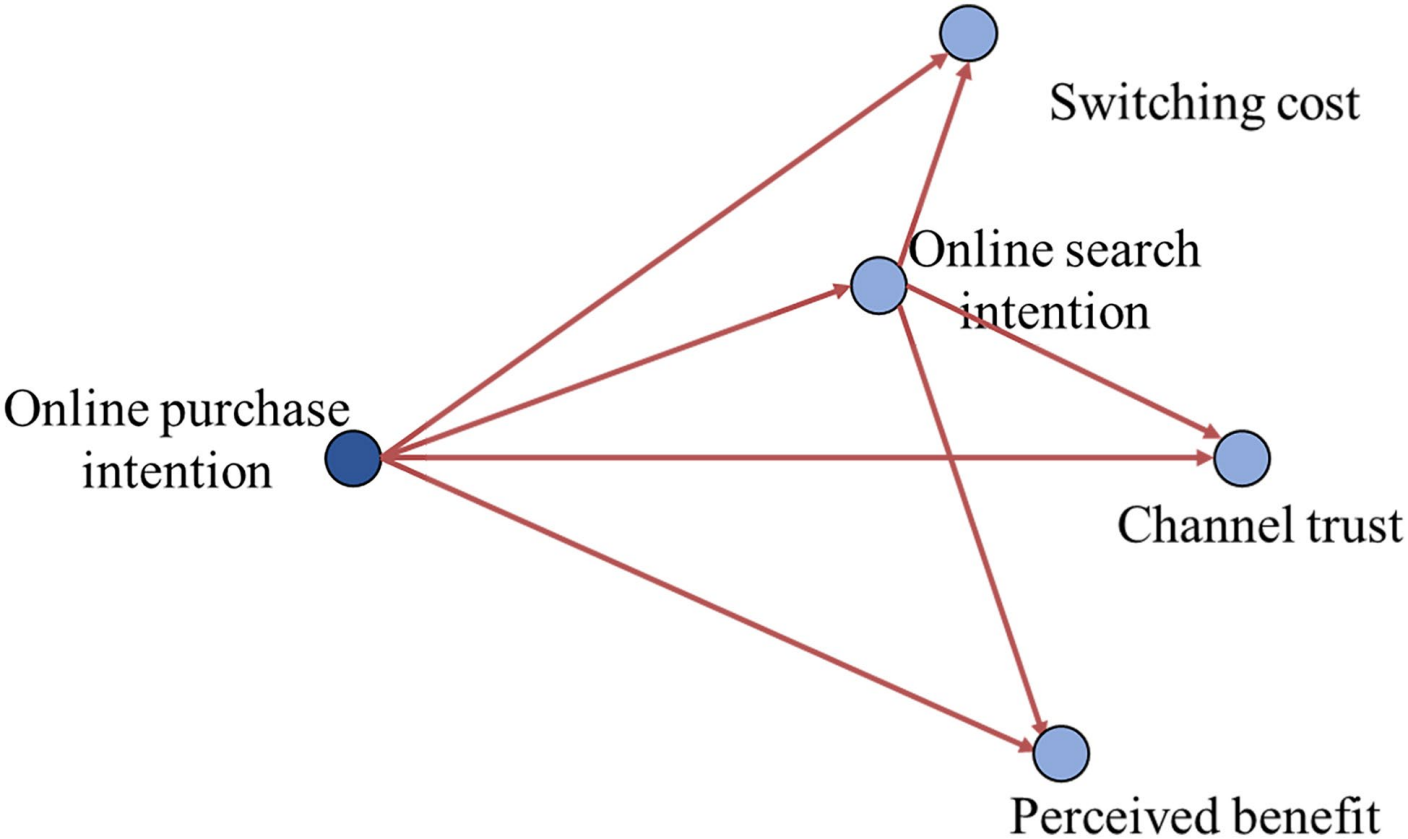
Bayesian network model of consumers’ willingness to choose channels.

We used SPSS modeler 18.0 to construct the TAN Bayesian network based on clustering data with the maximum likelihood method, as shown in Figure 4. We can see that purchase intention is the parent node of transfer cost, perceived benefit, search intention, and channel trust, indicating that purchase intention is affected by these four latent variables from the constructed Bayesian network structure. In addition, online search intention is the parent node of transfer cost, perceived benefit, and channel trust, indicating that online search intention is also affected by these three variables. The influence of online search intention on online purchase intention depends not only on itself but also on the perceived benefits of online purchase, channel trust, and channel transfer cost.

#### Bayesian Prediction

According to the constructed Bayesian network, the prediction of online search intention and purchase intention in different states can be obtained from switching costs, channel trust, and perceived benefit, as shown in Table 6. As can be seen from the tables, with the changes in switching costs, channel trust, and perceived benefit along the high-medium-low (simply called H-M-L), online search and purchase intentions change positively. Due to the low search cost of online channels, consumers tend to search for product information online after generating a shopping demand. Table 6A shows that the higher the perceived cost caused by the transfer of online search to offline purchase, the stronger consumers’ intention to choose purchase, indicating that the switching costs plays a positive role in the locking of online channels. From Table 6B, we can see that the state of “high” search intention and purchase intention changes positively with the change of channel trust from high to middle to low. Meanwhile, from Table 6C, it can be seen that, with the decrease of consumers’ perception of purchase benefit, the decreasing probability of high purchase intention is more obvious than that of high search intention, indicating that purchase benefit has a greater impact on online purchase intention. Table 6D shows that, when consumers’ willingness to search online is low, their willingness to buy online is also very low, indicating that consumers are less likely to choose the path of offline search–online direct purchase.

**Table 6:** (A) Bayesian inference of switching cost in different state, (B) Bayesian inference of channel trust in different states, (C) Bayesian inference of perceived interests in different states, and (D) Bayesian inference of search intention in different states.

(A)						
States	OSI			OPI		
	H	M	L	H	M	L
H	0.503	0.392	0.105	0.421	0.467	0.113
M	0.396	0.422	0.182	0.353	0.451	0.196
L	0.308	0.453	0.239	0.223	0.574	0.203
**(B)**						
H	0.574	0.383	0.043	0.458	0.485	0.057
M	0.455	0.437	0.108	0.241	0.551	0.209
L	0.248	0.545	0.207	0.157	0.471	0.373
**(C)**						
H	0.403	0.466	0.131	0.611	0.269	0.119
M	0.296	0.486	0.218	0.355	0.558	0.087
L	0.285	0.439	0.277	0.077	0.807	0.115
**States**	**(D)** **OPI**						
	H		M		L	
H	0.436		0.482		0.082	
M	0.221		0.581		0.198	
L	0.039		0.269		0.629	

#### Bayesian Diagnosis

Bayesian diagnosis is the reverse operation of Bayesian reasoning; that is, the state of the independent variables is obtained through the state of the dependent variables. The following tables show the Bayesian diagnosis of search intention, purchase benefit, online trust, and switching costs given the purchase intention of the parent node. Table 7A shows the conditional probability set of search intention under the condition of a given parent node of purchase intention. It can be seen from Table 7A that search intention changes positively with the change in the H-M-L intention, and when the online purchase intention is clear, consumers have a high probability of choosing online search product information, indicating that online channels have a certain channel lock.

**Table 7:** (A) Conditional probability table of search intention and (B) conditional probability of search intention and purchase intention.

(A)										
Parent node		Probability								
OPI		H			M			L		
H		0.683			0.311			0.006		
M		0.469			0.507			0.024		
L		0.253			0.549			0.198		
**(B)**										
**Parent node**		**PB**			**SC**			**CT**		
OSI	OPI	H	M	L	H	M	L	H	M	L
H	H	0.784	0.198	0.018	0.602	0.293	0.106	0.609	0.366	0.024
H	M	0.721	0.265	0.015	0.449	0.449	0.103	0.427	0.507	0.066
H	L	0.646	0.302	0.052	0.391	0.435	0.174	0.131	0.826	0.044
M	H	0.482	0.429	0.089	0.304	0.536	0.161	0.518	0.411	0.071
M	M	0.341	0.599	0.061	0.319	0.605	0.075	0.347	0.558	0.095
M	L	0.335	0.574	0.071	0.417	0.558	0.025	0.175	0.617	0.208
L	H	0.201	0.687	0.111	0.432	0.312	0.256	0.341	0.406	0.253
L	M	0.143	0.843	0.014	0.286	0.571	0.143	0.143	0.714	0.143
L	L	0.056	0.833	0.111	0.222	0.722	0.056	0.057	0.499	0.444

Table 7B shows the set of conditional probabilities of switching costs, perceived benefit, and channel trust under the conditions of search intention and purchase intention. It can be seen from Table 7B that, with the H-M-L change of search intention and purchase intention, the probability of switching costs, perceived benefit, and channel trust gradually decreases. When the search intention is in the state of “high,” with the change of H-M-L purchase intention, the probability of a “high” perceived benefit is not obvious, which indicates that perceived benefit is an important reason to attract consumers to choose an online channel to buy products, while the probability of perceived benefit, transfer cost, and channel trust being “medium” and “low” decreases at first and then increases. When the search intention is in the “middle” state, with the change of purchase intention from high to low, the change of channel trust to “high” is more obvious. This shows that whether consumers choose to buy products directly online depends to a large extent on the degree of trust of they have in the channel, and enterprises that carry out online retail business can attract consumers to online channels by improving consumers’ trust in online channels.

## Discussion

Combining the empirical ability of SEM and the predictive and diagnostic ability of Bayesian networks, we analyzed the factors influencing consumers’ online search and purchase intention in multi-retail channels as well as the relationship between these factors. The results showed that as: (1) Consumers’ perceived benefits, channel trust, and switching cost have a positive impact on search intention, and consumers’ trust in online channels is the main factor driving their choice of online search, this result is consistent with the results found in the previous studies (e.g., Hajli, 2015; Martin et al., 2015; Reimer and Benkenstein, 2016; Zhao et al., 2017). (2) Consumers’ perceived benefits, channel trust, and switching cost have a positive impact on purchase intention, and the main factor for attracting consumers to choose online product purchasing is the perceived benefit factor, the greater the perceived benefits, the stronger the consumers’ willingness to buy the products (e.g., Martin et al., 2015; van der Lans et al., 2016). (3) Consumers’ willingness to search online also affects their willingness to buy online, and this result is consistent with the results found in the previous studies (e.g., Ngwe et al., 2019; Zhai et al., 2019); therefore, guiding consumers to search for products will increase purchasing probability of sold products. (4) When channel trust reaches a certain level, online channels have a certain channel lock, that is, consumers will choose the path of online search–online purchase, and channel switching cost also has a positive effect on the online channel lock, this is because the higher the perception of switching cost, the less likely it is for consumers to search for product information in one channel and purchase products in another channel (e.g., Anderson and Simester, 2013; Stan et al., 2013). (5) According to the Bayesian network diagnosis, search intention can adversely affect consumers’ perceived benefit, channel trust, and switching cost, and purchase intention can adversely affect consumers’ perceived benefit, channel trust, switching cost, and search intention.

This research provides new ideas on the research methods of consumer channel selection. The existing research on consumer channel choice is mostly qualitatively based on theory or empirical analysis of the causal relationship between variables with the help of statistical software (e.g., Huang et al., 2016; Elida et al., 2019), while ignoring the in-depth discussion of the complex interrelationships between variables. This article proposes a combination of structural equation modeling and Bayesian network research methods to explore the variables and complex relationships that affect consumers’ willingness to choose online shopping channels, and in-depth analysis of the attributes of online channels, with a view to further enriching consumer channel choice behaviors related research.

For retailers carrying out online retail business, analyzing the influencing factors of consumers’ online choice under multiple channels helps to better satisfy consumers’ channel preference, thus increasing the probability of interaction between retailers and consumers, improving consumers’ channel stickiness, and reducing enterprise service costs. Therefore, this study has important practical implication for solving the problem of ineffective online channel operation after traditional retail enterprises adopt multi-channel retail strategy.

## Suggestions

According to the above research conclusions, we provide the following marketing suggestions for online retailers and company with an online business.

First, it is important to give attention to value marketing and strengthen customer stickiness. Online retailing as an important part of the new retail environment, and the continuous low-price strategy has been unable to retain consumers over the long term. Retailers need to balance the relationship between price and cost. Improving the price-to-performance ratio of products and the quality of distribution service is crucial to enhance the customer experience and maintain brand image. In addition, retailers can promote product updates, discount activities, brand value images and other information to customers through official accounts, well-known bloggers, and other ways to improve consumer loyalty.

Second, online retailers should focus on content marketing and improving customer attention. In the multi-channel retail environment, consumers have more independent choice of information search, and more vivid content is very important when consumers search and make purchase decisions. In addition to the e-commerce platforms, retailers could also make use of the emerging business infrastructure to provide convenient and quick product search channels, such as Mini Programs, official accounts or life accounts, to present products or brands in the form of text, pictures, short videos, and live broadcasts to attract consumers through multiple channels and increase consumer attention through multiple means. In addition, for different consumer groups, differentiated content marketing according to the positioning of the brand can also yield twice the results with half the effort in terms of attracting consumers’ attention.

Third, online retailers must engage in honest marketing and enhance the reputation of their brands. Trust is the key factor that supports the success of online retailing. The more consumers trust in the channel, the more likely they are to have positive search and shopping intention. Retailers can improve their credibility through the evaluation and certification of third-party sellers or with the help of consumers’ trust in well-known brands. Moreover, they can use credit mechanisms, such as “commitment+guarantee,” to allay consumers’ shopping concerns. This will impact the shopping procedures of consumers and help to win their trust.

## Limitation

In studying consumers’ willingness to choose channels under multiple retail channels, we considered their willingness to search and purchase through online channels, but we did not further compare online channels with offline channels. Future research will further refine channel selection factors, such as channel attributes. In addition, when analyzing consumers’ channel choice willingness in this study, we did not consider specific product types, because different product categories will affect consumers’ channel choice intention in the two-stage decision-making of searching information and purchasing products, future research could consider dividing different product categories or introducing other factors that affect consumer preferences to conduct research on consumers’ willingness to choose channels.

## Data Availability Statement

The raw data supporting the conclusions of this article will be made available by the authors, without undue reservation.

## Ethics Statement

Ethical review and approval was not required for the study on human participants in accordance with the local legislation and institutional requirements. Written informed consent for participation was not required for this study in accordance with the national legislation and the institutional requirements. Written informed consent was implied *via* completion of the survey.

## Author Contributions

All authors listed have made a substantial, direct and intellectual contribution to the work, and approved it for publication.

## Funding

This research has been funded by the National Social Science Fund of China under grant no. 20CGL004, the Social Science Foundation of the Chinese Education Commission under grant no. 15XJA630003, and the Doctor Foundation of Chongqing University of Posts and Telecommunications under grant no. A2015-20.

## Conflict of Interest

The authors declare that the research was conducted in the absence of any commercial or financial relationships that could be construed as a potential conflict of interest.

## Publisher’s Note

All claims expressed in this article are solely those of the authors and do not necessarily represent those of their affiliated organizations, or those of the publisher, the editors and the reviewers. Any product that may be evaluated in this article, or claim that may be made by its manufacturer, is not guaranteed or endorsed by the publisher.

## References

[ref1] Akbarzadeh M. Moghimbeigi A. Morris N. Daneshpour M. Mahjub H. Soltanian A. (2019). A Bayesian structural equation model in general pedigree data analysis. Stat. Anal. Data Min. 12, 404–411. doi: 10.1002/sam.11434

[ref2] Anderson E. Simester D. (2013). Advertising in a competitive market: the role of product standards, customer learning, and switching costs. J. Mark. Res. 50, 489–504. doi: 10.1509/jmr.11.0538

[ref3] Balasubramanian S. Raghunathan R. Mahajan V. (2010). Consumers in a multichannel environment: product utility, process utility, and channel choice. J. Int. Mark. 19, 12–30. doi: 10.1002/dir.20032

[ref4] Balladares G. Miralles F. Kennett C. (2016). “The role of perceived risk in online information search and pre-purchase alternative evaluation of products with significant experiential attributes,” in Strategic Innovative Marketing. eds. Kavoura A. Sakas D. Tomaras P. (Switzerland: Springer), 44–53.

[ref5] Burnham T. A. Frels J. K. Mahajan V. (2003). Consumer switching costs: a typology, antecedents, and consequences. J. Acad. Mark. Sci. 31, 109–126. doi: 10.1177/0092070302250897

[ref6] Chang H. H. Wong K. H. Li S. Y. (2017). Applying push-pull-mooring to investigate channel switching behaviors: M-shopping self-efficacy and switching costs as moderators. Electron. Commer. Res. Appl. 24, 50–67. doi: 10.1016/j.elerap.2017.06.002

[ref7] Chen-ran G. E. (2020). Brand sentiment, customer interaction and consumer purchasing behavior. Int. J. New Dev. Educ. 2, 22–23.

[ref8] Chickering D. M. (2002). Learning equivalence classes of Bayesian-network structures. J. Mach. Learn. Res. 2, 150–157.

[ref9] Costa e Silva S. Duarte P. Machado J. C. Martins C. M. (2020). Cause-related marketing in online environment: the role of brand-cause fit, perceived value, and trust. Int. Rev. Public Nonprofit. Mark. 17, 135–157. doi: 10.1007/s12208-019-00237-z

[ref10] Elida T. Rahardjo W. Raharjo A. Sukirman E. (2019). Online shopping: what factors determine consumers to buy? Manage. Stud. 7, 238–246. doi: 10.17265/2328-2185/2019.03.007

[ref11] Gupta A. K. Shukla A. V. (2019). Online retail format choice behavior of Indian customers for reasoned purchase: a cultural perspective. J. Int. Consum. Mark. 31, 469–491. doi: 10.1080/08961530.2019.1611518

[ref12] Hajli N. (2015). Social commerce constructs and consumer’s intention to buy. Int. J. Inf. Manag. 35, 183–191. doi: 10.1016/j.ijinfomgt.2014.12.005

[ref13] Haubl G. Trifts V. (2000). Consumer decision making in online shopping environments: the effects of interactive decision aids. Mark. Sci. 19, 4–21. doi: 10.1287/mksc.19.1.4.15178

[ref14] Holloway B. B. Wang S. Parish J. T. (2005). The role of cumulative online purchasing experience in service recovery management. J. Interact. Mark. 19, 54–66. doi: 10.1002/dir.20043

[ref15] Huang L. Lu X. Ba S. (2016). An empirical study of the cross-channel effects between web and mobile shopping channels. Inf. Manag. 53, 265–278. doi: 10.1016/j.im.2015.10.006

[ref16] Khan S. A. Liang Y. Shahzad S. (2015). An empirical study of perceived factors affecting customer satisfaction to re-purchase intention in online stores in China. J. Serv. Sci. Manag. 8, 291–305. doi: 10.4236/jssm.2015.83032

[ref17] King R. A. Racherla P. Bush V. D. (2014). What we know and don’t know about online word-of-mouth: are view and synthesis of the literature. J. Int. Mark. 28, 167–183. doi: 10.1016/j.intmar.2014.02.001

[ref18] Kuan H. H. Bock G. W. (2007). Trust Transference in Brick and Click Retailers: An Investigation of the Before-Online-Visit Phase. Netherlands: Elsevier Science Publishers B. V.

[ref19] Lee K. C. Chung N. Lee S. (2011). Exploring the influence of personal schema on trust transfer and switching costs in brick-and-click bookstores. Inf. Manag. 48, 364–370. doi: 10.1016/j.im.2011.09.002

[ref20] Lee C. K. Kim J. Kim J. S. (2018). Impact of a gaming company’s CSR on residents ‘perceived benefits, quality of life, and support. Tour. Manag. 64, 281–290. doi: 10.1016/j.tourman.2017.09.002

[ref21] Martin J. Mortimer G. Andrews L. (2015). Re-examining online customer experience to include purchase frequency and perceived risk. J. Retail. Consum. Serv. 25, 81–95. doi: 10.1016/j.jretconser.2015.03.008

[ref22] Ngwe D. Ferreira K. J. Teixeira T. (2019). The impact of increasing search frictions on online shopping behavior: evidence from a field experiment. J. Mark. Res. 56, 944–959. doi: 10.1177/0022243719865516

[ref23] Reimer T. Benkenstein M. (2016). When good WOM hurts and bad WOM gains: the effect of untrustworthy online reviews. J. Bus. Res. 69, 5993–6001. doi: 10.1016/j.jbusres.2016.05.014

[ref24] Schneider P. J. Zielke S. (2020). Searching offline and buying online – an analysis of showrooming forms and segments. J. Retail. Consum. Serv. 52, 52–55. doi: 10.1016/j.jretconser.2019.101919

[ref25] Singh S. Jang S. (2020). Search, purchase, and satisfaction in a multiple-channel environment: how have mobile devices changed consumer behaviors? J. Retail. Consum. Serv. 10, 10–16. doi: 10.1016/j.jretconser.2020.102200

[ref26] Singh S. Swait J. (2017). Channels for search and purchase: does mobile internet matter? J. Retail. Consum. Serv. 39, 123–134. doi: 10.1016/j.jretconser.2017.05.014

[ref27] Song X. Y. Chen F. Lu Z. H. (2013). A Bayesian semiparametric dynamic two-level structural equation model for analyzing non-normal longitudinal data. J. Multivar. Anal. 121, 87–108. doi: 10.1016/j.jmva.2013.06.001

[ref28] Song X. Y. Lee S. Y. (2008). A Bayesian approach for analyzing hierarchical data with missing outcomes through structural equation models. Struct. Equ. Model. 15, 272–300. doi: 10.1080/10705510801922472

[ref29] Song X. Y. Xia Y. M. Pan J. H. Lee S.-Y. (2011). Model comparison of Bayesian semiparametric and parametric structural equation models. Struct. Equ. Model. 18, 55–72. doi: 10.1080/10705511.2011.532720

[ref30] Stan V. Caemmerer B. Cattan-Jallet R. (2013). Customer loyalty development: the role of switching costs. Metal. Int. 18, 95–99. doi: 10.3390/ma6010184

[ref31] Stewart D. W. Qin Z. (2013). Internet marketing, business models, and public policy. J. Public Policy Mark. 19, 287–296. doi: 10.1509/jppm.19.2.287.17125

[ref32] Thietart R.-A. (2015). Strategy dynamics: agency, path dependency, and self-organized emergence. Strateg. Manag. J. 37, 774–792. doi: 10.1002/smj.2368

[ref33] Tien I. Kiureghian A. D. (2016). Algorithms for Bayesian network modeling and reliability assessment of infrastructure systems. Reliab. Eng. Syst. Saf. 156, 134–147. doi: 10.1016/j.ress.2016.07.022

[ref34] van der Lans R. van Everdingen Y. Melnyk V. (2016). What to stress, to whom and where? A cross-country investigation of the effects of perceived brand benefits on buying intentions. Int. J. Res. Mark. 33, 924–943. doi: 10.1016/j.ijresmar.2016.05.002

[ref35] Verhoef P. C. Neslin S. A. Vroomen B. (2007). Multichannel customer management: understanding the research-shopper phenomenon. Int. J. Res. Mark. 24, 129–148. doi: 10.1016/j.ijresmar.2006.11.002

[ref36] Wang M. Sun L.-L. Hou J.-D. (2021). How emotional interaction affects purchase intention in social commerce: the role of perceived usefulness and product type. Psychol. Res. Behav. Manag. 14, 467–481. doi: 10.2147/PRBM.S301286, PMID: 33889034 PMC8055276

[ref37] Zhai Q. Cao J. Zhen F. (2019). Relationship between online shopping and store shopping in the shopping process: empirical study for search goods and experience goods in Nanjing, China. Transp. Res. Rec. 2673, 38–47. doi: 10.1177/0361198119851751

[ref38] Zhao S. Chen L. (2021). Exploring residents’ purchase intention of green housings in China: an extended perspective of perceived value. Int. J. Environ. Res. Public Health 18:4074. doi: 10.3390/ijerph18084074, PMID: 33924311 PMC8069697

[ref39] Zhao X. Deng S. Zhou Y. (2017). The impact of reference effects on online purchase intention of agricultural products: the moderating role of consumers’ food safety consciousness. Internet Res. 27, 233–255. doi: 10.1108/IntR-03-2016-0082

